# Nanocrystals for Improved Drug Delivery of Dexamethasone in Skin Investigated by EPR Spectroscopy

**DOI:** 10.3390/pharmaceutics12050400

**Published:** 2020-04-27

**Authors:** Silke B. Lohan, Siavash Saeidpour, Miriam Colombo, Sven Staufenbiel, Michael Unbehauen, Amanuel Wolde-Kidan, Roland R. Netz, Roland Bodmeier, Rainer Haag, Christian Teutloff, Robert Bittl, Martina C. Meinke

**Affiliations:** 1 Department of Dermatology, Venerology and Allergology, Center of Experimental and Applied Cutaneous Physiology, Charité—Universitätsmedizin Berlin, Corporate Member of Freie Universität Berlin, Humboldt-Universität zu Berlin, and Berlin Institute of Health, 10117 Berlin, Germany; martina.meinke@charite.de; 2 Fachbereich Physik, Freie Universität Berlin, 14195 Berlin, Germany; siavash.saeidpour@uni-rostock.de (S.S.); woldeaman@zedat.fu-berlin.de (A.W.-K.); rnetz@physik.fu-berlin.de (R.R.N.); christian.teutloff@fu-berlin.de (C.T.); robert.bittl@fu-berlin.de (R.B.); 3 Pharmazeutische Technologie, Institut für Pharmazie, Freie Universität Berlin, 14195 Berlin, Germany; colo.miriam90@gmail.com (M.C.); sven.staufenbiel@fu-berlin.de (S.S.); bodmeier@zedat.fu-berlin.de (R.B.); 4 Institut für Chemie und Biochemie, Freie Universität Berlin, 14195 Berlin, Germany; unbehaumich@zedat.fu-berlin.de (M.U.); haag@chemie.fu-berlin.de (R.H.)

**Keywords:** skin penetration, nanocarriers, nanocrystals, penetration kinetics, 1D general diffusion equation, electron paramagnetic resonance (EPR) spectroscopy

## Abstract

Nanocrystals represent an improvement over the traditional nanocarriers for dermal application, providing the advantages of 100% drug loading, a large surface area, increased adhesion, and the potential for hair follicle targeting. To investigate their advantage for drug delivery, compared to a base cream formulation, dexamethasone (Dx), a synthetic glucocorticoid frequently used for the treatment of inflammatory skin diseases, was covalently linked with the paramagnetic probe 3-(carboxy)-2,2,5,5-tetramethyl-1-pyrrolidinyloxy (PCA) to DxPCA. To investigate the penetration efficiency between these two vehicles, electron paramagnetic resonance (EPR) spectroscopy was used, which allows the quantification of a spin-labeled drug in different skin layers and the monitoring of the drug release. The penetration behavior in excised healthy and barrier-disrupted porcine skin was monitored by EPR, and subsequently analyzed using a numerical diffusion model. As a result, diffusion constants and free energy values in the different layers of the skin were identified for both formulations. Dx-nanocrystals showed a significantly increased drug amount that penetrated into viable epidermis and dermis of intact (factor 3) and barrier-disrupted skin (factor 2.1) compared to the base cream formulation. Furthermore, the observed fast delivery of the spin-labeled drug into the skin (80% DxPCA within 30 min) and a successive release from the aggregate unit into the viable tissue makes these nanocrystals very attractive for clinical applications.

## 1. Introduction

The stratum corneum (*SC*) represents the greatest challenge for the penetration of active substances into the viable skin. It is composed of 10 to 20 cell layers of coherent, coreless corneocytes, which are embedded in a lipid-enriched intercellular space, where ceramides, free fatty acids, and cholesterol constitute the main part, forming bilayer structures of 4 to 5 nm thickness. Located in the uppermost layer of the epidermis, the SC thus represents the primary barrier between the organism and the environment [1,2].

The topical application of drugs incorporated into a cream formulation is the most frequently used form of therapy for the treatment of skin diseases [3]. Impairments in the integrity of the horny layer, which are characteristic for inflammatory skin diseases, facilitate epicutaneous treatments [4,5]; nevertheless, each skin disease has a different severity of integrity disorder, making a standard skin care approach difficult. Also, the efficiency of direct penetration of the lipid-rich SC is crucially determined by the exact properties of the diffusing drug, since it has been shown that the chemical structure affects solute interactions with lipid bilayers [6].

The incorporation of medication into a vehicle, such as nano-sized carrier systems, is intended to improve the penetration of drugs into intact and also barrier-disrupted skin. A targeted dosage can be carried out, improving the effectiveness of the treatment of affected skin areas and thus minimizing side effects [7,8]. In recent years, various carrier systems, such as liposomes, nano-emulsions, or polymer-based particles, have been developed, depending on the chemical and physical properties of the drug of interest [9,10,11]. Often they have limited success, due to the insufficient drug load.

Electron paramagnetic resonance (EPR) spectroscopy enables the localization of active substances and drug analogues in nanocarrier systems using a covalently bound spin label, which can report on microenvironment properties like polarity and viscosity [12,13,14]. Furthermore, the release of spin probes from nanocarriers and their skin penetration efficiency can be investigated, and an absolute quantification of them can be performed as well [15].

Dexamethasone (Dx) is an artificial glucocorticoid, and one of the most important drugs to suppress the body’s immune system and to control inflammation processes. It inhibits the formation of inflammatory substances (prostaglandins), as well as the production of new immune cells, suppressing further immune reactions [16,17]. Dx has been covalently labeled with the spin probe 3-(carboxy)-2,2,5,5-tetramethyl-1-pyrrolidinyloxy (PCA) to form DxPXA, which has been well-established as a model drug for ex vivo penetration studies by EPR. First, the loading capacity with DxPCA and its localization in carrier system was characterized, followed by skin penetration investigations on ex vivo skin. During these investigations, Dx and DxPCA were found to be poorly soluble in aqueous solution [18,19,20].

Our own EPR studies have shown that dendritic core–multi-shell (CMS) particles are efficient drug delivery systems for the topical application of hydrophilic substances to the skin. In direct comparison to lipid-based invasomes, CMS nanocarriers have shown increased penetration, especially in the upper layers of the SC [21]. In addition, improved cutaneous penetration of the model drug DxPCA loaded in CMS particles was demonstrated vs. DxPCA dissolved in aqueous solution [19]. A comparison between a base cream formulation and solid lipid nanoparticles (SLNs) exhibited a generally improved penetration efficiency of DxPCA into the SC for SLN. After 24 h, a more than two-fold higher penetration efficiency for SLNs was observed. Furthermore, a combination of different spectroscopic and microscopic methods exhibited a reservoir function within the SC and the hair follicles for SLNs [18].

Nanocrystals are another promising carrier system. They are formulations that enable increased bioavailability of sparingly soluble active ingredients. In contrast to other carriers, they consist of pure active substance—i.e., they have 100% drug loading. Due to their “nano size”, they have a higher kinetic saturation solubility [22] and a higher dissolution rate compared to normal powders. After dermal application, an increased concentration gradient between formulation and skin, and thus an increased passive penetration is obtained [23]. Nanocrystals can be used to improve dermal penetration processes, as has been demonstrated with the model drug curcumin [24].

Nanocrystals represent a promising alternative to the carrier systems, which have already been investigated extensively by EPR, as mentioned above. Their main advantage lies in their high loading capacity. Therefore, the aim of this study was to analyze this carrier system and to check whether the epicutaneous application of Dx to skin could be further improved.

The penetration of the model drug DxPCA into intact and barrier-disrupted ex vivo porcine skin was examined using EPR spectroscopy. A DxPCA nanocrystal suspension and a base cream formulation with the same drug concentration were compared. The temporal profile of the DxPCA penetration, its release kinetics into the skin, as well as the results from the data-based model for intact and barrier-disrupted skin were investigated and critically compared, in order to finally assess the penetration efficiency of the nanocrystals.

## 2. Materials and Methods

### 2.1. Chemicals

For the preparation of spin-labeled DxPCA, chemicals from Merck Chemicals GmbH (Darmstadt, Germany) were used, if not listed separately.

All chemicals for the base cream production (except DxPCA) were received from Caesar and Loretz GmbH (Hilden, Germany).

### 2.2. Preparation of Spin-Labeled Dexamethasone (DxPCA)

To enable the analysis of the penetration kinetics of the glucocorticoid dexamethasone (Dx) by EPR spectroscopy, Dx was covalently labeled with the spin probe PCA. This spin probe is stable in the skin and not immediately reduced by the microenvironment [25,26]. DxPCA was synthesized by heating to reflux Dx with PCA, 4-(Dimethyl)-aminopyridine and 1-Ethyl-3-(3-dimethylaminopropyl) carbodiimide in dichloromethane solution for 3 h with subsequent column–chromatographic purification. Finally, DxPCA had a labeling efficiency of ~80%, measured by EPR [25]. This powder (Figure S1) was used for the preparation of the DxPCA nanocrystals and a DxPCA base cream formulation.

### 2.3. Preparation of DxPCA Nanocrystals

Nanocrystals of PCA-labeled dexamethasone were prepared by wet bead milling. The drug was dispersed into 0.5% (*w/v*) poloxamer 407 solution (Kolliphor 407, BASF, Ludwigshafen, Germany) and homogenized by an Ultra Turrax (T25, IKA-Werke GmbH, Staufen, Germany) at 20,500 rpm for 30 s. Ultra-purified water, purified by a Milli-Q apparatus (Millipore GmbH, Darmstadt, Germany), was used. The suspension was afterwards added to a 100 mL Erlenmeyer flask containing 0.1–0.2 mm zirconium beads (ratio suspension/beads = 1:3) (SiLibeads, Sigmund Lindner GmbH, Warmensteinach, Germany) and milled for 3 h under magnetic stirring at ~800 rpm. The Erlenmeyer was kept in an ice bath to ensure low product temperatures during milling. The nanosuspension was separated from the beads by filtration through a filter paper with a pore size of ~45 µm (Sartorius AG, Goettingen, Germany). Finally, the nanosuspension was filtered through a 1.2 µm filter to exclude big particles (Whatman GE Healthcare Ltd., Buckinghamshire, United Kingdom).

The real drug content was measured after filtration by UV spectroscopy (Agilent HP 8453, Agilent Technologies Inc., Santa Clara, CA, United States). Subsequently, the suspension was diluted with a 0.5% (*w/v*) poloxamer 407 solution to obtain the desired final drug concentration (0.05% (*w/w*)), which was confirmed by UV analysis. The nanosuspension particle size was measured by photon correlation spectroscopy (PCS) (Zetasizer Nano ZS, Malvern Instruments Ltd., Malvern, United Kingdom).

### 2.4. Preparation of 0.05% (w/W) DxPCA in Base Cream (Cremor Basalis)

The batch size was 25 g and was prepared with mortar and pestle. The composition of cremor basalis is (% m/m) 4.0 glycerol monostearate 60, 6.0 cetyl alcohol, 7.5 Miglyol 812, 25.5 white soft paraffin, 7.0 Tagat S2, 10.0 propylene glycol, and 40.0 ultrapurified Milli-Q water (Millipore GmbH, Darmstadt, Germany) (Table 1). The preparation of cremor basalis was as follows: the oil phase (glycerol monostearate 60, cetyl alcohol, Miglyol 812, white soft paraffin) and the water phase (Tagat S2, water) were homogenized separately at 70 °C in a water bath. Subsequently, the water phase was dispersed into the oil phase and stirred until room temperature was reached. Afterwards, propylene glycol and evaporated water were added homogenously. The incorporation of coarse DxPCA into cremor basalis was completed by dispersing DxPCA in an equivalent amount of Miglyol 812. An equivalent amount of cremor basalis (the final concentration of Miglyol 812 in cremor basalis was kept at 7.5% m/m) was homogenized with this mixture. This step was repeated until batch size was reached.

### 2.5. EPR Spectroscopy

The EPR experiments were performed on a X-band EPR spectrometer (Bruker Elexsys E500, BioSpin GmbH, Karlsruhe, Germany) at room temperature. A SHQ resonator (E4122011SHQE-W1, Bruker BioSpin GmbH, Karlsruhe, Germany) was used for spin quantification with the following parameter settings: frequency = 9.8 GHz, central magnetic field = 350 mT, magnetic field sweep width = 20 mT, modulation frequency = 100 kHz, modulation amplitude = 0.2 mT, and attenuation = 22 dB. A TMHS resonator and a rapid scan unit (E2044500TMHS, Bruker BioSpin GmbH, Karlsruhe, Germany) were used for penetration studies with the following parameter settings: frequency = 9.5 GHz, central magnetic field = 350 mT, magnetic field sweep width = 20 mT, modulation frequency = 100 kHz, modulation amplitude = 0.3 mT, attenuation = 10 dB, and scans = 50,000. The analysis of all EPR data was performed using the Bruker device control software Xepr (Bruker Biospin, Karlsruhe, Germany).

#### 2.5.1. Spin Quantification of the Vehicles Loaded with DxPCA

The amount of spin-labeled DxPCA within the used vehicles (base cream, nanocrystals) was determined as previously described [18]. Both the nanocrystal suspension and the base cream formulation were diluted at a ratio of 1:10 with DMSO (Sigma Aldrich, Darmstadt, Germany) before spin quantification was performed using glass capillaries (Hirschmann Laborgeräte GmbH & Co. KG, Eberstadt Germany) sized 1.2 mm/1.0 mm (outer diameter/inner diameter).

The absolute spin concentrations and the spin amounts were measured and calculated in triplicate.

#### 2.5.2. Penetration Study of DxPCA into Intact and Barrier-Disrupted Skin

For modeling the penetration kinetics of spin-labeled Dx into intact and barrier-disrupted skin, porcine skin, which is an accepted model for human skin, has been used [27], with the approval of the Veterinary Office Berlin Treptow-Köpenick. The skin was delivered by a local butcher on the day of slaughter and purified as previously described [28]. To simulate a barrier disorder of the skin, the procedure of “tape stripping” was applied [29,30,31]. In order to simulate barrier-disrupted skin, as it occurs in inflammatory skin diseases, 50 tapes (Tesa film No. 5529, Beiersdorf, Hamburg, Germany) were removed before the topical application of the vehicles. The tapes were removed from the skin as described previously [32,33]. With the removal of 50 tapes, the skin barrier is specifically disturbed, but 2–3 µm of the stratum corneum remain [31]. Afterwards, 20 μL/cm^2^ (12 µg/ cm^2^ ± 1 µg/ cm^2^) of the respective formulation was applied onto the skin (intact/ barrier-disrupted) and was evenly distributed by vibration massage for 2 min (Rehaforum Medical GmbH, Elmshorn, Germany), followed by an incubation at 32 °C in a chamber with 100% humidity to avoid dehydration of the skin. The penetration kinetics of DxPCA was analyzed by EPR 30 min, 1 h, and 4 h after topical application. After the respective penetration time, the uppermost layer of the skin (400 µm) was excised using a dermatome (Aesculap Acculan 3Ti Dermatom, Aesculap-Werke AG, Tuttlingen, Germany). Skin sections 0.5 × 1.5 cm in size were placed into a tissue cell (ER 162TC-Q, Bruker, Bruker Biospin GmbH Karlsruhe, Germany) and measured at ambient temperature (21 °C).

For the evaluation of the amount of DxPCA within the different skin layers, the skin with supernatant (remaining DxPCA which is not completely uptaken by the skin) after the respective incubation period was used as initial value; therefore, the size and the thickness of the analyzed skin samples in combination with the double integration of the EPR spectrum was used for the absolute spin quantification.

All measurements were performed in triplicate on different porcine ear skin samples.

### 2.6. Numerical Modeling

The one-dimensional (1D) generalized diffusion equation is used to describe the time-dependent skin penetration of the drug. The inhomogeneous skin structure is modeled in terms of depth-dependent diffusivity *D*(*z*) and a free-energy landscapes *F*(*z*). The diffusivity describes the local mobility of the drug molecules, while the free energy describes the local affinity of the drug to the environment. The diffusion equation can be written compactly as

(1) $$\frac{\partial c\left(z,t\right)}{\partial t}=\frac{\partial }{\partial z}\left[D\left(z\right)e^{-\beta F\left(z\right)}\frac{\partial }{\partial z}\left(c\left(z,t\right)e^{\beta F\left(z\right)}\right)\right],$$

where *c*(*z,t*) is the concentration at depth *z* and time *t*. As introduced in previous studies [34,35], the numerical solution to Equation (1) was used to extract diffusivity profiles and local free energy values from the measured concentration data. In light of the reduced spatial resolution of the present data, it was assumed that the values for *D*(*z*) and *F*(*z*) have piecewise constant values within the formulation applied onto the skin, the stratum corneum and the epidermis. The integrated concentration in these three regions from the numerically calculated concentration profiles was then fitted to the experimentally measured values and used to determine the optimal D and F values. Good agreement between experimental data and numerically computed DxPCA concentrations was obtained with deviations in terms of the root mean squared relative error ranging from σ = 1.5%–4%.

For the discretization of the diffusion equation, a total of 99 discretization sites were used, with 33 sites per region at variable discretization widths from Δ*z* = 0.1–10 µm, depending on the sample size. The optimization of the parameters was performed using the non-linear trust region method implemented in Python’s *scipy* package [36]. Error bars were estimated from averaging over different numerical solutions that exhibited errors deviating less than 10% from the optimal numerical solution. Since only integrated concentration profiles are fitted to the experimental data, and the experimental measurements were already close to the stationary distribution, uncertainties for the extracted parameters are relatively large.

In the case of experiments performed with DxPCA nanocrystal suspensions, one could in principle augment Equation (1) to also include the DxPCA release kinetics from the crystals and model the DxPCA fraction present in form of nanocrystals and the DxPCA fraction released into solution separately. Since the experimental data do not allow us to determine all necessary kinetic parameters uniquely, such an augmented diffusion equation could not be used. The obtained values for the diffusion constant and energy differences thus represent effective parameters that describe both the DxPCA bound in the nanocrystals and the free DxPCA in solution.

### 2.7. Permeability Coefficients

A quantity commonly used to evaluate the efficiency of penetration is the so-called permeability coefficient *P*, which is defined in terms of the flux *J* that is produced by a difference in concentration at both sides of a barrier:

(2) $$P\left(z_{1},z_{2}\right)\;=\frac{J}{c\left(z_{1}\right)-c(z_{2})}$$

where *c* is the concentration at the left *(z_1_)* or right *(z_2_)* side of the barrier.

Using the determined values for the diffusion constants and the free energy, the permeability of the skin samples can be computed as

(3) $$\;\frac{1}{P_{tot}}=\frac{1}{P_{SC}}+\frac{1}{P_{epi}}=\frac{L_{SC}}{D_{SC}K_{SC}}+\frac{L_{epi}}{D_{epi}K_{epi}}$$

where *D_i_* denotes the diffusion constant; *K_i_* = exp(−*F_i_*/*k_B_T*) is the partition coefficient, computed from the free energy in the respective regions relative to the outer medium; and *L_i_* is the length of the segments (*SC*: stratum corneum, *epi*: epidermis). This equation for the permeability coefficient is based on the assumption that the medium embedding the diffusional barrier (the skin sample in this case) is the same on both sides (at *z_1_* and *z_2_*).

### 2.8. Statistics

For all statistical analyses, SPSS for Windows (SPSS Inc., Chicago, IL, United States) was used. Differences in mean values were analyzed using repeated measures (RM) analysis of variance (ANOVA) (one-way and two-way). A value of *p* ≤ 0.05 was considered as significant.

## 3. Results

### 3.1. Investigation of the Mobility of DxPCA in the Vehicles by EPR

DxPCA was mixed into a base cream and used for a nanocrystal suspension, which had a particle size of 260–290 nm. Both vehicles exhibit a spin concentration of (7.3 ± 0.6) × 10^14^ mm^3^, which is equivalent to a concentration of 1 mM.

**Figure 1:**
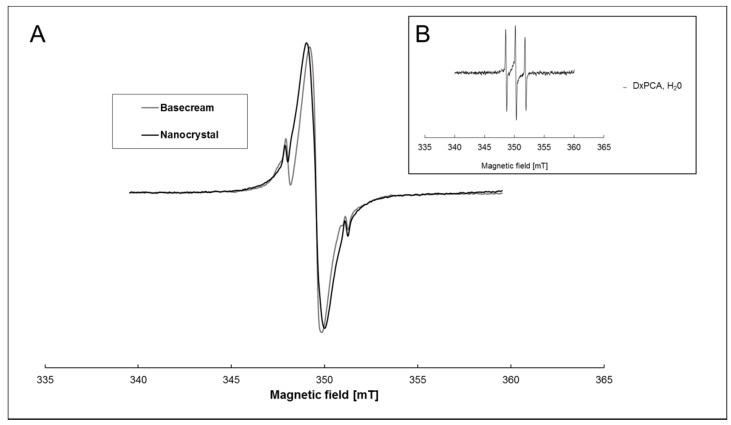
X-band electron paramagnetic resonance (EPR) spectra of dexamethasone–3-Carboxy-proxyl (DxPCA). (**A**) 1 mM of DxPCA dissolved in a base cream (grey line) and in a nanocrystal formulation (black line) at ambient temperature. Both spectra are normalized to the maximum height of the middle peak. (**B**) DxPCA (90 µM) dissolved in an aqueous solution [19].

The EPR spectra of both vehicles are visually similar (Figure 1A). These spectra are a superposition of an intense central line and a weak, typical three-line spectrum of a nitroxide in solution (DxPCA in water shown in Figure 1B for reference). The strong and approximately 10 G broad central line arises from spin–spin coupling between the densely packed DxPCA molecules within the nanocrystal formulation and the presence of DxPCA aggregates within the base cream. The weak three-line spectrum in Figure 1A originates from dissolved/free DxPCA. Only the low- and high-field lines of the three-line spectrum are visible in Figure 1A, while the middle line is obscured by the strong central line. Hence, Figure 1A shows that different states of DxPCA coexist in both formulations. For comparison, DxPCA in an aqueous solution (unbound) shows three narrow peaks, illustrating high mobility (Figure 1B) [19].

### 3.2. Penetration of DxPCA into Healthy and Barrier-Disrupted Excised Porcine Skin, as Analyzed by EPR Spectroscopy

To investigate the penetration efficiency and kinetics of DxPCA dissolved in a base cream formulation, as well as in a nanocrystal formulation into intact and barrier-disrupted porcine ear skin, EPR measurements at X-band were performed.

**Figure 2:**
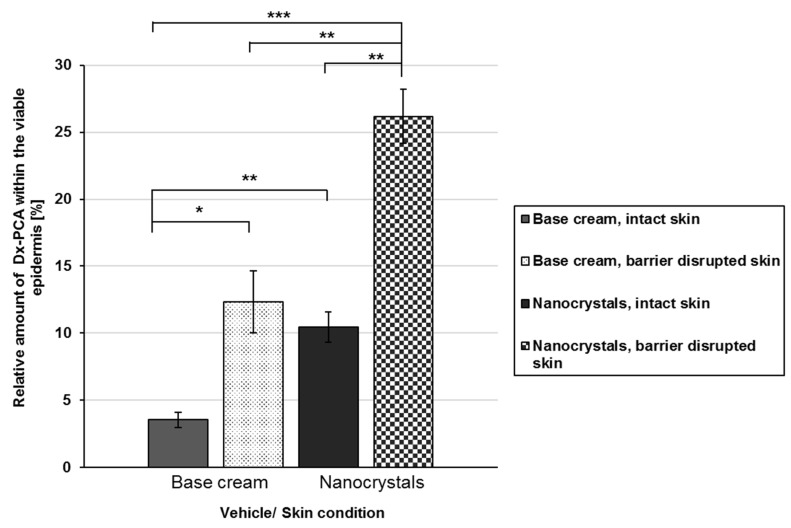
Relative amount of DxPCA within the viable epidermis for intact (painted bars) and barrier-disrupted (shaded bars) skin after 4 h of incubation time. The data are calculated relative to the total amount applied, realized by measuring the skin sample with supernatant (remaining DxPCA that is not completely taken up by the skin) by EPR spectroscopy immediately after the incubation time; Mean ± SEM, * *p* ≤ 0.05, ** *p* ≤ 0.01, *** *p* ≤ 0.001.

**Figure 3:**
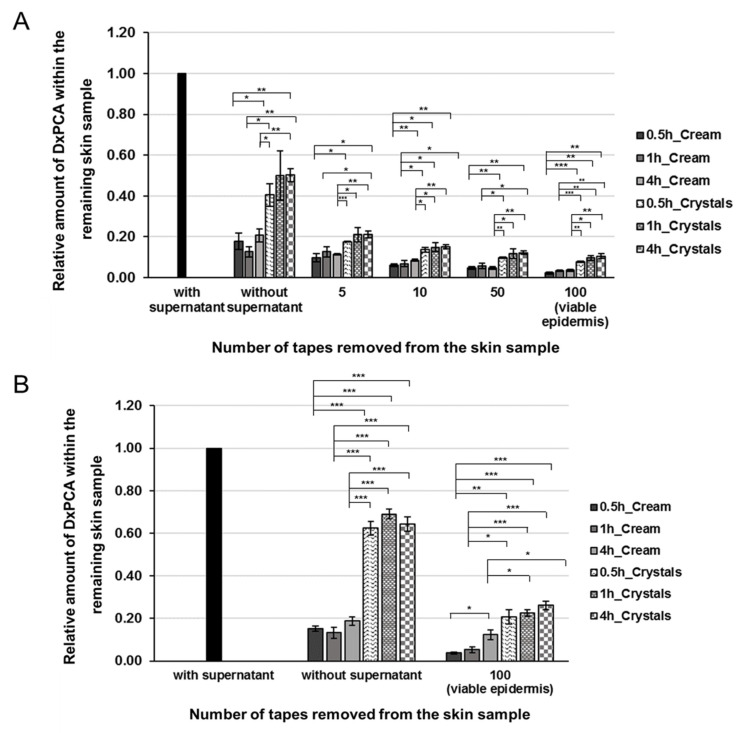
Distribution profile of DxPCA in the remaining intact (**A**) and barrier-disrupted (**B**) skin samples after the topical application of a base cream (monochrome, 1mM), a nanocrystal suspension (pattern design, 1mM), and various incubation times (0.5, 1 and 4h). To analyze the distribution of DxPCA in intact skin different amounts of the stratum corneum (SC) were removed by tape stripping and the remaining skin samples were analyzed. To analyze the amount of DxPCA within the viable epidermis of barrier-disrupted skin, an additional 50 tapes were removed after the respective incubation time; for the intact skin, 100 tapes were removed; Mean ± SEM, * *p* ≤ 0.05, ** *p* ≤ 0.01, *** *p* ≤ 0.001.

The EPR investigations demonstrate that the penetration of the corticosteroid can be enhanced by three independent parameters, the formulation itself, the penetration time, and the skin condition (Figure 2 and Figure 3).

The usage of nanocrystals increases the local concentration gradient for both intact and barrier-disrupted skin significantly, so that higher amounts of DxPCA reached the viable epidermis compared to the base cream formulation (Figure 2). For intact skin, nanocrystals increased the amount of the drug within the viable epidermis by a factor of 3 in comparison to the cream formulation (3.6% vs. 10.5%). An inclusion of the skin’s health status indicates that a disruption of the skin barrier enhanced the permeability for both vehicles (base cream, nanocrystals). An intra-individual comparison of the penetration shows an increase of almost 3.5 times, and for the nanocrystals 2.1 times, between intact and barrier-disrupted skin for the base cream. An inter-individual comparison demonstrates that nanocrystals promote the penetration of DxPCA into the viable epidermis to a greater extent. Already, in intact skin, a significant increase in the DxPCA-concentration can be detected. Thus, a barrier disruption is required to obtain comparable concentrations of DxPCA with the base cream formulation, as in intact skin, with the nanocrystals in the viable epidermis (Figure 2).

However, not only the selection of the formulation itself, but also an extension of the penetration time showed positive effects on the penetration behavior of DxPCA into intact or barrier-disrupted skin. For both the base cream and nanocrystal formulation, an extension of the penetration time from 30 min up to 4 h increased the amount of drug that penetrated into the individual skin layers and the viable epidermis (Figure 3). For intact skin, it was shown that a major proportion of DxPCA is present in the viable epidermis after 30 min. An extension of the penetration time increased the already existing DxPCA levels, especially for the base cream formulation (factor of 1.8) (Figure 3A). For barrier-disturbed skin, an extension of the penetration time to 4 h increases the DxPCA content by a factor of 3 in the case of the base cream formulation, and for the nanocrystals a 4 h incubation improves the penetration by a smaller factor (1.26) (Figure 3B). It is clearly shown that nanocrystals have significantly reduced the required penetration time for DxPCA into the skin in comparison to the base cream formulation.

The absolute spin quantification illustrates, for the nanocrystals, an increased penetration of DxPCA into the skin vs. base cream formulation (Table 2).

### 3.3. Release of DxPCA from the Vehicle into Healthy and Barrier-Disrupted Excised Porcine Skin, as Shown by EPR

**Figure 4:**
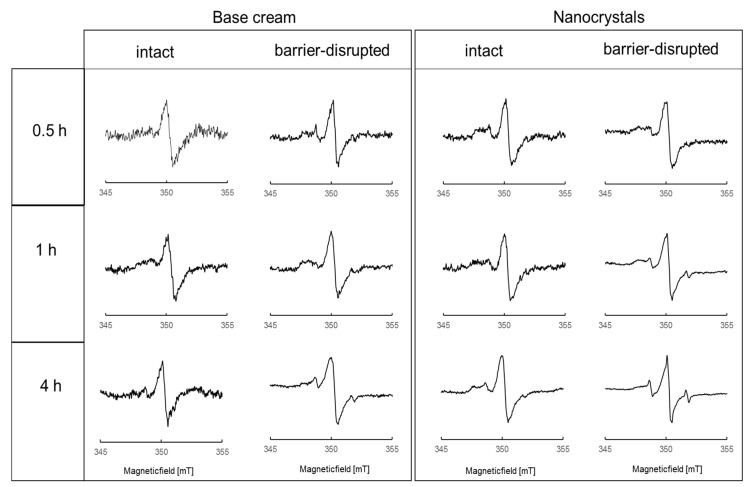
Normalized X-Band EPR spectra of spin-labeled Dx within the viable epidermis after the topical application of a base cream formulation (left column) and nanocrystals (right column) to intact and barrier-disrupted skin and penetration times of 0.5, 1.0, and 4.0 h.

Figure 4 shows the X-band EPR spectra of spin-labeled Dx within the viable epidermis after incubation times of 0.5, 1.0, and 4.0 h. All X-band EPR spectra were normalized to the same peak intensity of the central peak.

The spectral shape of DxPCA in skin (Figure 4) is significantly different from that seen in both formulations and in water (Figure 1). The central peak of all EPR spectra in the skin is much narrower than in the formulations (Figure 1A). In skin, the formation of a clear intensity left of the central peak is visible around 348 mT; it is best discernable in the nanocrystal spectra (Figure 4, right columns). This intensity can be assigned to dissolved but partially immobilized DxPCA in the absence of significant spin–spin interaction. For both formulations, this immobilized DxPCA fraction is the main species. Highly mobile DxPCA, giving rise to the sharp three-line spectral component, similar to PCA in water (Figure 1B), is the minority. The absence of spin–spin coupling in this majority species shows that the vast amount of DxPCA is present in the skin as dissolved species.

Extension of the penetration time increases the amount of mobile DxPCA compared to the immobilized fraction in barrier-disrupted skin for both formulations. Application of DxPCA by the nanocrystal formulation on skin results in more and faster penetration of the drug into different skin layers, in comparison to the base cream formulation.

For the assessment of the microenvironment of mobile DxPCA, the hyperfine coupling constant (*hfc*) was determined from the obtained EPR spectra. This parameter indicates whether the mobile drug molecules are in a more lipophilic or aqueous environment, and can be obtained by evaluating the splitting of the lines in the three-line part of the spectrum. This splitting gives an *hfc* value of 45.8 MHz for DxPCA in both vehicles before topical application on skin, which corresponds to DxPCA in an aqueous environment (45.1 MHz for DxPCA in water [18]). After topical application and 4 h penetration time, the *hfc* values changed to 41.9 MHz and 41.7 MHz for base cream and nanocrystals, respectively, within the viable epidermis. These *hfc* values are very close to the 40.6 MHz [18] reported for DxPCA dissolved in the amphiphilic DMSO. Thus, the mobile DxPCA fraction, after application by both vehicles within the viable epidermis, is very likely in an amphiphilic environment [37].

### 3.4. Data-Based Modeling of the Penetration Kinetics of DxPCA into Healthy and Barrier-Disrupted Skin

**Figure 5:**
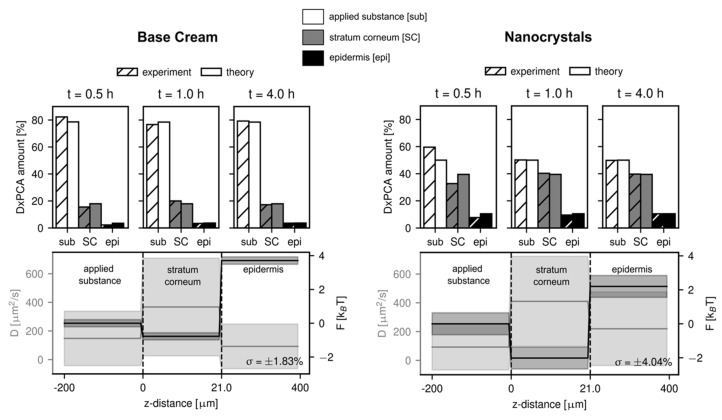
Results of the numerical analysis for the penetration of DxPCA in the two formulations in the case of healthy skin. A root mean squared error of below 5% is obtained for all analyses. An increased partitioning of DxPCA into the SC is observed for the nanocrystal formulation, apparent in the lower energy value in the SC for the nanocrystal suspension (a partition coefficient of *K*(SC/sub) = 7.4 was obtained, compared to *K*(SC/sub) = 2.2 for the base cream formulation). Shaded areas indicated the estimated error for the diffusion constant and free energy.

Results of the numerical analysis based on the one-dimensional general diffusion equation can give further insight into the experimental data. Here we analyze the diffusion constant and free energy in three different regions: the DxPCA formulation applied to the skin (termed applied substance), the SC, and the epidermis. Figure 5 shows the results for the two DxPCA formulations penetrating into healthy skin. As explained in the Methods section, the estimation of the diffusion constants in the three different regions is difficult, due to the relatively long incubation times before the measurement started. The upper panels of Figure 5 show that the penetration process is already almost completed after 30 min. It is thus hard to evaluate the significance of the slight increase of the DxPCA mobility in the SC compared to the applied substance, as observed for both the base cream and nanocrystal formulation (see lower panels of Figure 5). The values of the obtained diffusion constants lie, however, in the range of those expected based on previous analyses [34,35], even though the skin samples were treated differently. The free energy landscape looks similar for both DxPCA formulations; however, a significantly lower value of the free energy in the SC is observed in the case of the Dx nanocrystals. This effect of lowering the free energy level in the SC by the nanocrystals is even more pronounced in the case of the barrier-disrupted skin samples (see Figure S2). The energy barrier towards the lower skin layers consisting of the epidermis and the dermis is of the same magnitude for both, the base cream and the nanocrystal suspension and is in agreement with previous studies [34,35]. As the free energy landscape determines the partition coefficient *K* between the different regions and the applied substance as *K* = exp(−*F*/*k_B_T*), where *k_B_T* denotes the thermal energy, this indicates a preferred partitioning of DxPCA into the SC, for both formulations, with a stronger effect for the nanocrystal formulation.

### 3.5. Permeability Coefficients for Intact and Barrier-Disrupted Skin

**Figure 6:**
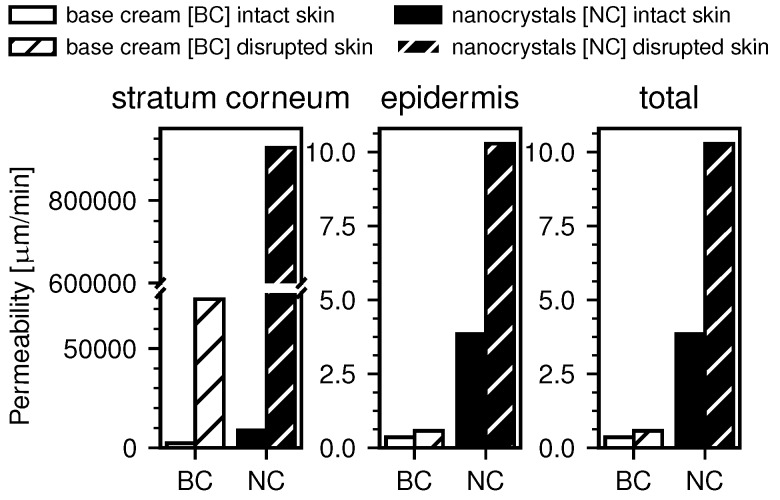
Computed permeability coefficients from the numerical data. The permeability in the SC is the highest, due to the free energy minimum here and the smaller length scale. All obtained permeabilities are higher for the case of the nanocrystal formulation compared to the base cream. Also, barrier-disrupted skin shows higher permeability compared to intact skin.

Using the previously determined values for the diffusion constants and the free energy, the permeability coefficients for intact and barrier-disrupted skin for both DxPCA formulations were determined based on Equation (3) in the Methods section (see Figure 6).

Figure 6 shows the computed permeability coefficients in the SC and the epidermis, as well as the total permeability coefficients. It is apparent that the permeability in the SC is several magnitudes larger than in the epidermis for all measurements. This is due to the previously discussed minimum in the free energy in the SC and the resulting partitioning of the DxPCA, but also because of the much smaller thickness of the SC. The total permeability is, however, dominated by the smallest permeability in the inverse sum, which is obtained for the epidermis. For both, the barrier-disrupted and the intact skin samples, the obtained permeability coefficients are significantly higher in the case of the nanocrystal formulation. Additionally, barrier disrupted skin shows increased permeability over intact skin.

## 4. Discussion

The efficiency of topically applied drugs to penetrate into the skin depends on their chemical and physical properties [38], the vehicle used [39], and the skin condition [40]. Only the consideration of all these factors can achieve an efficient penetration and thus therapeutic effectiveness in the skin. Several skin models have been established for determining the penetration kinetics of drugs into the skin [40]. Due to intra- and inter-individual variability of the skin models, only limited standardized data are available [40,41]. In chronic inflammatory skin diseases, such as atopic dermatitis, the barrier function of the skin is disturbed and thus weakened [42]. In this study, the penetration kinetics of a common therapeutic agent for atopic eczema—spin-labeled dexamethasone, a synthetic glucocorticoid which is frequently used for the treatment of inflammatory skin diseases—was analyzed and loaded into two different transport vehicles: a base cream and a nanocrystal formulation. Its penetration efficiency and concentration levels in intact and barrier-disrupted porcine ear skin, which is a suitable model for human skin [43,44], were investigated by EPR spectroscopy. To induce an artificial barrier disruption, conventional tape stripping [30,45] was applied. To support the experimental research results, penetration models for DxPCA into skin were created (1D general diffusion equation) that took the kinetic penetration profile of intact and barrier-disturbed skin into account.

Using spin-labeled DxPCA, it is possible to investigate the microenvironment of the drug, track its release from the base cream or nanocrystal dispersion, and follow its penetration into the skin in detail. For example, a pH-dependent release of DxPCA could already be shown in solution and in the skin by changes in the EPR spectra and related magnetic parameters [46]. Also, a slow release of the spin label from a carrier system in skin can be traced with the EPR spectra [15]. Investigations by Saeidpour et al. showed that the chemical properties of Dx do not change drastically when PCA is covalently bound (Dx logP: 1.83, DxPCA logP: 1.89) [47]. Moreover, comparable penetration results have been obtained from other groups using unlabeled Dx in their penetration studies [34,35]. Therefore, microenvironment investigations and penetration kinetics analysis into skin from DxPCA are assumed to be comparable to unlabeled Dx. Furthermore, the labelling of the drug Dx with the spin probe PCA enables the absolute quantification of its penetration into the individual skin layers [18], which allows the assessment of the efficiency of drug penetration into skin (Table 2 and Figure 3).

DxPCA was mixed into a standard base cream formulation and a nanocrystal suspension. In both vehicles, the EPR spectra of the spin-labeled drug indicate a two-phase system. The EPR spectra show a strong broadening of the central peak due to spin–spin coupling of the densely packed model drug DxPCA in the nanocrystals and aggregates in the base cream (Figure 1). The low and high field peaks of the DxPCA spectra were only slightly present (minor free fraction). In aqueous solution, DxPCA displays three narrow lines, which reflects high mobility [19,47]. The EPR spectra allow the estimate that the vast majority (≥98%) of the spin-labeled Dx molecules are aggregated, and only a small percentage is dissolved, not only in the nanocrystals but in the base cream. The aggregation in the base cream could indicate the presence of microcrystals or amorphous structures. Interestingly, both formulations could not be clearly distinguished from each other by EPR spectra, and comparable penetration results would be assumed. 

After topical application of these two formulations onto skin, the DxPCA *hfc* value of the highly mobile fraction decreased from ~45.8 MHz (before topical application) to ~41.9 (base cream) and ~41.7 MHz (nanocrystals), comparable to the 40.6 MHz in DMSO, indicating an amphiphilic microenvironment for the mobile fraction of DxPCA [18,47] within the viable epidermis. However, the majority of DxPCA is found in dissolved form in the viable epidermis, in a microenvironment of low mobility.

The penetration of spin-labeled Dx into intact and barrier-disrupted skin was investigated after three different penetration times (0.5, 1.0, and 4.0 h). A comparison between intact and barrier-disrupted skin shows an increased penetration of DxPCA into the disturbed skin for both vehicles. In the individual skin layers up to the viable epidermis, an increased drug distribution could be determined (Figure 2 and Figure 3). After 4 h, the amount of DxPCA was significantly increased by a factor of ~3 for the base cream; for nanocrystals, a significant enhancement of factor ~2 could be achieved (intact vs. barrier-disturbed skin) (Figure 2). Only a barrier disorder enables the base cream to deliver a comparable amount of DxPCA into the viable epidermis, as can be achieved with nanocrystals and intact skin (Figure 2). Nanocrystals appear to provide a better surface-to-volume ratio compared to the incorporation of Dx powder in a cream formulation, resulting in a better dissolution of this nanoparticle structure upon application onto skin, thus ensuring a higher local concentration gradient. Thus, nano-sized crystals display an effective vehicle for the transport of DxPCA into both intact and barrier-disturbed skin. 

Compared to previous EPR studies, in which nano-transport systems were also investigated, nanocrystals show a significant improvement for the DxPCA transport into the intact skin. Compared to other particles, nanocrystals show a higher loading of the active ingredient DxPCA (approaching the limit of 100% particle loading): nanocrystal formulation (~1mM) > pH sensitive Eudragit particles (530 µM) [46] > SLN (200 µM) [18] > CMS (90 µM) [19]. Because nanocrystals facilitate the release of DxPCA into the viable skin, the use of them in the treatment of inflammatory skin lesions should be preferable. Due to their higher loading, nanocrystals enhance the penetration of DXPCA into intact skin by a factor of 3.8 (after a penetration time of 4 h) compared to SLN [18]. 

However, not only the loading of a vehicle, but also its physical-chemical properties importantly influence the permeability of the active ingredient into the skin. Although the load of the pH-sensitive Eudragit nanoparticles is half of that of nanocrystals, they show a better penetration of DxPCA into both intact skin (factor 6) and barrier-disturbed skin (factor 2) compared to a base cream formulation. The possibility of a targeted release at an increased pH value (>5.5) in deeper skin layers, as well as in the hair follicle, enables increased drug delivery into the viable skin vs. base cream [46] and nanocrystals. Thus, ideally nanocrystals providing the highest concentration of active ingredient in the skin would be combined with a targeted release at the treatment site. This could be made possible by encapsulating these particles into, for example, pH sensitive particles. 

A successive increase in the drug concentration was measurable in the individual skin layers. In the first 30 min, most of DxPCA penetrated the skin, which was only slightly increased by a longer penetration time (Figure 3). The barrier function of the skin is mainly located in the middle of the SC, at approximately 8 µm, due to maximum values for trans-conformation and lateral packing order of the intercellular lipids [48]. The lateral packing of lipids is more disordered on the surface and in the deeper parts of the SC, which may be associated with a reduced skin barrier function. These areas could serve as a reservoir [49]. The cells of the SC, the corneocytes, are embedded in a structured, multilamellar lipid matrix consisting mainly of ceramides, cholesterol, and free fatty acids [50]. Our results confirm that the barrier function of the skin is decisive for the penetration kinetics of active substances [51,52]; the penetration time contributes only to a small part.

The change in the spectral shape of DxPCA for base cream and nanocrystals demonstrates that an extended penetration time of 4 h promotes the amount of a minor, highly mobile DxPCA fraction in the viable epidermis (Figure 4). Both formulations show a main DxPCA fraction with restricted rotational motion in skin. From our investigations, it is impossible to conclude which form is more relevant for therapeutic applications. In general, steroid hormones can induce their effect by binding to intracellular receptors located in the cytosol (glucocorticoid receptor (GR)), but also to membrane-associated receptors, which are a specific isoform or a modification of the classical GRs [53].

The increased penetration observed for the DxPCA nanocrystals compared to the base cream formulation is due to several effects. First, the nanocrystal formulation shows a decrease of the free energy in the SC compared to the base cream formulation, as indicated by the diffusion model (Figure 5). In terms of the partition coefficient between the DxPCA formulation applied to the skin and the SC, the base cream formulation leads to a value of *K*(SC/formulation) = 2.2, compared to *K*(SC/formulation) = 7.4 for the DxPCA nanocrystal suspension. Obviously, the nanocrystal suspension considerably enhances the efficiency of the SC´s reservoir capacity. However, also the computed permeability coefficients show a higher value for the nanocrystal formulation compared to the DxPCA base cream (Figure 6). The total permeability of the skin sample is dominated by the value of the epidermis, which is significantly increased in the case of the nanocrystal formulation. This effect might be caused by the dissolution kinetics of the DxPCA nanocrystals. The analysis of the barrier-disrupted skin samples shows a higher permeation of DxPCA for both applied substances compared to the intact skin samples (see Figure S2 in the Supplementary Materials and Figure 6). In a recent study, tape stripping has been shown to affect only the diffusive barrier function of the skin, and not the equilibrium partitioning [35]. The results obtained in this work, however, do show an influence on the partitioning between the DxPCA solutions and the SC, with increased preference of DxPCA for the SC in the case of barrier-disrupted skin. This could be rationalized by the different treatments of the skin samples. In the referenced study, the skin was treated with a Dx dispersion containing ethanol [35,54], which is known to reduce the barrier function of the SC [55]. In the current study, however, neither the base cream nor the nanocrystal solution contained ethanol.

The topical treatment with, for instance, corticosteroids, represents the basis for inflammatory therapy approaches. Topical glucocorticoids act via nuclear receptors. After binding to such receptors, the gene expression of proteins that inhibit proinflammatory molecules in their activity and lead to vasoconstriction changes will be activated [56]. DxPCA dissolved in a cell lysate of secondary keratinocytes shows an *hfc* value of 42.1 MHz, and also restricted mobility like in skin (data not shown). In general, the penetration through the *SC* is necessary for drugs to execute a medical effect on the affected skin area. It can be assumed that DxPCA, after penetration into deeper skin layers, is stored in the intralipid matrix of the viable epidermis, or even absorbed by cells and localized in the cytoplasm, or bound to the glucocorticoid receptor itself [53,57,58]. Thus, not only the penetration into the viable epidermis, but also the mobility of the drug to reach the receptors is important for the medical effects. This study illustrates that both effects can be studied very effectively by EPR spectroscopy, because it allowsfor the analysis of the drug without destroying the skin.

## 5. Conclusions

In comparison to other drug carrier systems, nanocrystals represent an efficient vehicle for drugs with a low solubility in aqueous solutions. The dispersion of drug nanocrystals in liquid media leads to “nanosuspensions”, which consist of 0.05% (*w/w*) of the active DxPCA and enable an increased dissolution rate. This results in a fast and efficient release rate. 

The formulation itself, its active ingredient load, and the condition of the skin barrier have a strong influence on the penetration behavior of an active ingredient into the skin: a disturbed skin barrier in combination with nanoparticles as carrier system improves the penetration enormously vs. the base cream formulation. Nanocrystals promote the penetration of 80% DxPCA within the first 30 min. Additionally, the amount of a DxPCA fraction with higher mobility is more pronounced for nanocrystals than for the base cream formulation after a longer incubation time. 

## Figures and Tables

**Table 1:** Overview of the composition, with the respective concentration of each component of the cremor basilis (1) and the final drug-containing formulation (2); dexamethasone– 3-(carboxy)-2,2,5,5-tetramethyl-1-pyrrolidinyloxy (DxPCA).

**(1) Cremor Basalis DAC (German Pharmaceutics Codex)**	
**Component**	**Concentration (% Weight/Weight)**
glycerol monostearate 60	4.0
cetyl alcohol	6.0
medium-chain triglycerides (Miglyol 812)	7.5
white soft paraffin	25.5
Macrogol-20-glycerol-monostearate (Tagat S2)	7.0
propylene glycol	10.0
ultrapurified Milli-Q- water	40.0
**(2) Final Drug-Containing Formulation**	
**Component**	**Concentration (% Weight/Weight)**
DxPCA	0.05
Cremor basalis DAC	99.95

**Table 2:** Quantitative analysis of absolute spin concentration within the analyzed skin sample ([spins × 10^14^/cm^2^]) of DxPCA within the whole skin after the removal of the supernatant for the respective incubation time (corresponding to the applied DxPCA amount of 12 ± 1 µg/cm^2^).

		Incubation Time		
Applied Formulation	Skin Condition	0.5 h	1 h	4 h
Base cream	intact skin	10 ± 2	12 ± 2	18 ± 3
	barrier-disrupted skin	11 ± 1	10 ± 2	13 ± 1
Nanocrystals	intact skin	53 ± 9	61 ± 11	69 ± 6
	barrier-disrupted skin	85 ± 3	90 ± 1	84 ± 1

## Supplementary Materials

The following are available online at https://www.mdpi.com/1999-4923/12/5/400/s1. Figure S1. Structural formula of DxPCA. The spin label PCA is marked with a dotted circle. Figure S2. Numerical analysis of DxPCA penetration into barrier disrupted skin. Increased permeation compared to the healthy skin samples is observed and rationalized by an increased preference of DxPCA for the SC. The obtained partition coefficients K(SC/sub) = 7.2 for the base cream formulation and K(SC/sub) = 86.9 for the nanocrystals, display this increased affinity to the SC compared to the healthy skin samples. Additionally, the previously mentioned effect of the nanocrystals, leading to a stronger reservoir function of the SC is even more apparent for the disrupted skin samples. Shaded areas indicated the estimated error for the diffusion constant and free energy.
