# Supplementary material for: Nanocrystals for Improved Drug Delivery of Dexamethasone in Skin Investigated by EPR Spectroscopy
Source: Pharmaceutics. 2020 Apr 27;12(5):400. doi: 10.3390/pharmaceutics12050400 (PMC7284345; doi:10.3390/pharmaceutics12050400)
Supplement: Supplementary file 1 [file pharmaceutics-12-00400-s001.pdf]

pharmaceutics

Supplementary Materials: Nanocrystals for Improved Drug Delivery of Dexamethasone in Skin Investigated by EPR Spectroscopy

Silke B. Lohan, Siavash Saeidpour, Miriam Colombo, Sven Staufenbiel, Michael Unbehauen, Amanuel Wolde-Kidan, Roland R. Netz, Roland Bodmeier, Rainer Haag, Christian Teutloff, Robert Bittl and Martina C. Meinke

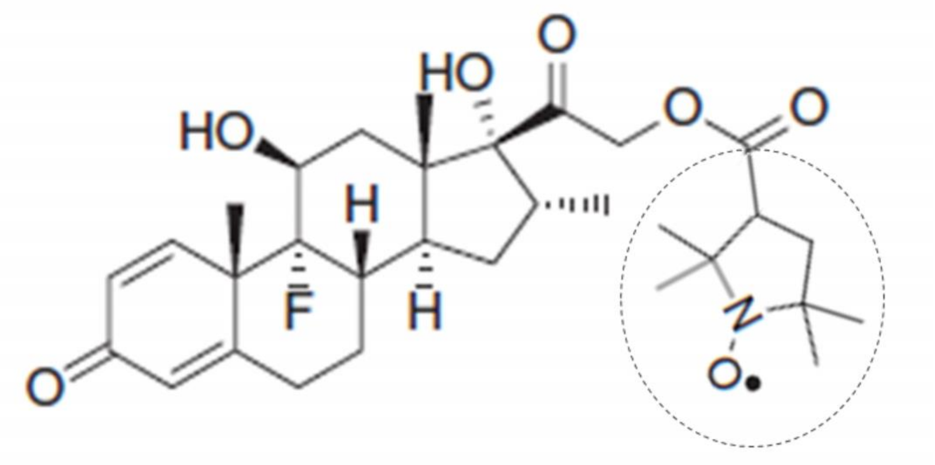

Figure S1. Structural formula of DxPCA. The spin label PCA is marked with a dotted circle.

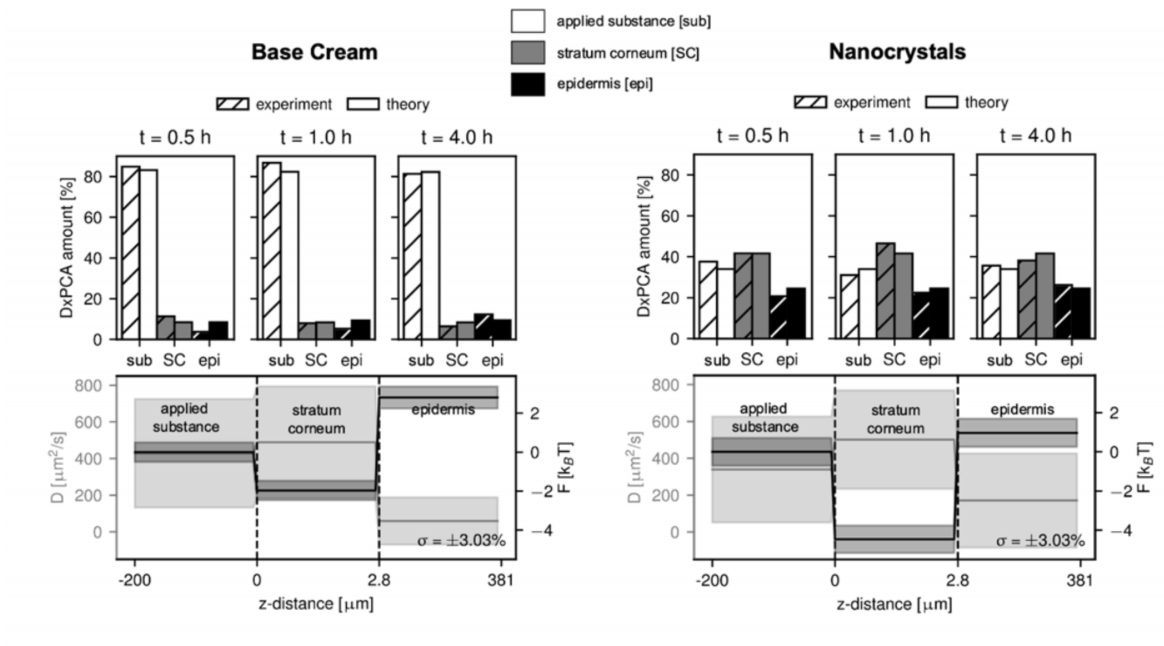

Figure S2. Numerical analysis of DxPCA penetration into barrier disrupted skin. Increased permeation compared to the healthy skin samples is observed and rationalized by an increased preference of DxPCA for the SC. The obtained partition coefficients K(SC/sub) = 7.2 for the base cream formulation and K(SC/sub) = 86.9 for the nanocrystals, display this increased affinity to the SC compared to the healthy skin samples. Additionally, the previously mentioned effect of the nanocrystals, leading to a stronger reservoir function of the SC is even more apparent for the disrupted skin samples. Shaded areas indicated the estimated error for the diffusion constant and free energy.
